# Lifetime Prevalence of Nonsuicidal Self‐Injury in Adolescents: Reliability of a Dichotomous Survey Item

**DOI:** 10.1002/mpr.70115

**Published:** 2026-10-21

**Authors:** Erik Aspeqvist, Hedvig Andersson, Örjan Dahlström, Laura Korhonen, Maria Zetterqvist

**Affiliations:** ^1^ Department of Biomedical and Clinical Sciences Center for Social and Affective Neuroscience Linköping University Linköping Sweden; ^2^ Department of Behavioral Sciences and Learning Linköping University Linköping Sweden; ^3^ Athletics Research Center Linköping University Linköping Sweden; ^4^ Clinical Department of Child and Adolescent Psychiatry in Linköping Region Östergötland Linköping Sweden; ^5^ Department of Biomedical and Clinical Sciences Barnafrid Swedish National Center on Violence Against Children Linköping University Linköping Sweden

**Keywords:** adolescents, lifetime prevalence, measurement, nonsuicidal self‐injury, reliability, survey

## Abstract

**Introduction:**

Nonsuicidal self‐injury (NSSI) is a widespread phenomenon among adolescents. Accurate estimates of prevalence rates when screening for NSSI are essential both in research and clinical settings. However, high variability raises the question of the reliability of self‐reported NSSI prevalence.

**Methods:**

The reliability of a dichotomous survey item on history of NSSI was investigated longitudinally using data from five time points across 16 months in a community sample (*N* = 253) of Swedish adolescents (age *M* = 14.21 years, SD = 0.56, 58.5% female).

**Results:**

About one in five participants (18.97%) changed their answer compared to their initial response. Of those who answered negatively on lifetime prevalence of NSSI at baseline, 8.95% later acknowledged NSSI, while 57.69% of those initially acknowledging NSSI later answered negatively. Fleiss' kappa across all five measurements indicated moderate agreement. Cohen's kappa between baseline and 12 months indicated moderate agreement.

**Conclusions:**

In this adolescent sample, the reliability of a single‐item question on NSSI history was moderate. More than half of those who initially acknowledged a history of NSSI answered negatively at a later time point. Interpretation of self‐reported lifetime prevalence rates of NSSI in adolescents using dichotomous survey items needs to be made cautiously.

## 1 Introduction

Nonsuicidal self‐injury (NSSI) is defined as the intentional self‐inflicted damage to bodily tissue performed without suicidal intent and for reasons not culturally sanctioned (International Society for the Study of Self‐Injury 2025). Estimates point to one in five adolescents engaging in NSSI (Muehlenkamp et al. 2012; Swannell et al. 2014), a rate that also seems to be rising (Gillies et al. 2018; Zetterqvist et al. 2021). There is a strong correlation with psychiatric disorders among adults with a history of NSSI (Liu 2023), and NSSI is associated with increased risk for suicidal behaviors (Kiekens et al. 2018). Further, more frequent NSSI during adolescence is linked to a range of negative outcomes in adulthood (Daukantaite, Lundh, et al. 2020). NSSI is thus a common and concerning mental health problem in adolescents.

In the epidemiology of mental disorders and mental health issues generally, lifetime prevalence, meaning the proportion of individuals who have ever experienced a certain phenomenon, is a highly used measure (Kessler et al. 2005; Merikangas et al. 2010). This is also true for the epidemiology of NSSI specifically (Staring et al. 2023; Xiao et al. 2022), even though it has been known for some time that retrospective lifetime prevalence measures are associated with several challenges. In adults, self‐reported history of self‐injury (with and without suicidal intent), for example, has been found to be a poor indicator of medically verified self‐injury and a poor predictor of future episodes (Borschmann et al. 2017). In adolescents, self‐reported history of NSSI results in false positives compared to semi‐structured interviews (Ross and Heath 2002). Further, agreement between child and parent reports of NSSI are typically poor (DeVille et al. 2020; Wichstrom and Wichstrom 2024). Self‐reported lifetime *frequency* can also be inconsistent in many cases, with respondents reporting lower frequencies at later measurements (Daukantaite, Lantto, et al. 2020).

In self‐report surveys, NSSI is usually measured using either single‐item methodology or behavioral checklists, with single‐item resulting in lower estimates than checklists (Muehlenkamp et al. 2012; Swannell et al. 2014). Single‐item measurement has been the slightly more common method (Swannell et al. 2014). An example of a single‐item question is the one used in the Self‐Injurious Thoughts and Behaviors Interview, short‐form, self‐report version (SITBI; Nock et al. 2007), with the wording “Have you ever actually engaged in NSSI?..” which is answered with “Yes” or “No”. Behavioral checklists, for example the Inventory of Statements about Self‐injury (Klonsky and Glenn 2009), typically feature a range of NSSI behaviors (e.g., cutting, biting, burning). Respondents indicate if they have engaged in one or several of the specified methods and define the frequency for each method over a specific time period (e.g., lifetime, 12 months or 6 months). In fact, when the same adolescents in community samples report NSSI prevalence in surveys using both methods, the prevalence doubles from single‐item to checklist (from 17.2% to 35.6% in Zetterqvist et al. 2013, and from 20.7% to 44.4% in Aspeqvist et al. 2024). The discrepancies between single‐item and checklist methodology potentially lead to false negatives and contribute to unreliable prevalence estimates. Furthermore, it has been shown that single‐item measurement alone can lead to systematic misrepresentation of different groups with a history of NSSI (Robinson and Wilson 2020). One such example is that more girls than boys acknowledge NSSI on the single item compared to checklist (Aspeqvist et al. 2024).

The challenges reviewed above are important to address, as accurate estimates and correct interpretations of prevalence rates in screening of NSSI in research and clinical settings are essential. Examining the reliability and validity of survey methodology could serve to strengthen the potential conclusions from epidemiological findings. However, while some results have been published earlier regarding the validity of types of questions cross‐sectionally (Aspeqvist et al. 2024; Robinson and Wilson 2020) and for self‐reported frequency (Daukantaite, Lantto, et al. 2020), little is known about the longitudinal reliability of dichotomous survey questions in the adolescent population.

In the present study, dichotomous reporting of NSSI was investigated in a sample of community adolescents with five measurements across 16 months. The main aim was to examine the reliability over time of self‐reported lifetime NSSI prevalence using single‐item methodology in a sample of Swedish community adolescents.

## 2 Materials and Methods

### 2.1 Procedure

Data in the present study were collected as part of a whole‐school prevention project focusing on mental health and NSSI among Swedish secondary school students. Six schools in Östergötland county were recruited to the project, and students in 7th and 8th grade were invited to participate. The participation rate was 25.3% of the eligible students. Data collection took place at the schools, during scheduled hours, with study staff present, at baseline (T1) and at follow‐ups after three (T2), six (T3), 12 (T4) and 14–16 (T5) months. Data were collected between January 2022 and June 2023. The recruitment and data collection procedures, as well as the interventions implemented in the whole‐school prevention project, have been described in detail in previously published papers (Aspeqvist et al. 2024, 2025). Schools were randomized to either an intervention group or a control group. Students at schools in the intervention group received interventions between T1 and T2, and students in the control schools received interventions between T3 and T4. All students, from both intervention and control groups, were included in the present study.

### 2.2 Ethics and Consent

Before participation, written informed consent from both caregivers and the students were required. The study was approved by the Swedish Ethical Review Authority (2021−01699, 2021–05049).

### 2.3 Participants

Out of the 266 adolescent participants at baseline, 13 were excluded because they did not participate in any follow‐up measurement, resulting in a final sample of 253 adolescents in the current study. Of these participants, 58.5% were female, 39.13% male and 2.37% non‐binary. The mean age was 14.21 years (SD = 0.56). See Table 1 for sample characteristics.

**TABLE 1:** Sample characteristics.

	Full sample (*N* = 253)	Participants with five complete measurements (*n* = 127)	Participants with complete measurements at baseline and 12‐months (*n* = 205)
Age (*M*, SD)	14.21 (0.56)	14.12 (0.57)	14.21 (0.58)
Gender			
Male (%)	39.13	40.16	37.07
Female (%)	58.5	58.27	60.49
Non‐binary (%)	2.37	1.57	2.44

### 2.4 Measures

#### 2.4.1 Demographic Information

At baseline, participants answered survey questions regarding background information, such as age and gender.

#### 2.4.2 Lifetime Prevalence of NSSI

Lifetime prevalence of NSSI was measured using the single‐item question “Have you ever actually engaged in purposely hurting yourself without wanting to die? (i.e., intentionally hurting yourself without wanting to die? e.g., cutting or burning yourself.)” from the SITBI‐SF‐SR (Nock et al. 2007).

### 2.5 Statistical Analysis

Fleiss' kappa (Fleiss 1971) for the dichotomous item was calculated taking into account all of the five measurements. Test‐retest reliability comparing the baseline and 12‐month follow‐up measurement was calculated using Cohen's kappa (Cohen 1960). Further, subgroups were identified based on participants consistently answering either “Yes”, “No”, or changing between either of those answers. Basic characteristics for these groups were calculated.

As participants received an intervention during the timeframe in which data were collected and the aim of the current study was to examine reliability of the NSSI single‐item rather than intervention effects, we also needed to investigate whether receiving the intervention was related to change in participants' responses on the lifetime NSSI prevalence item. To examine this, chi‐square tests were conducted. Specifically, the number of response changes between baseline and three months (T1–T2), baseline and six months (T1–T3), and six months and 12 months (T3–T4) were compared across the intervention and control groups. This was done as the intervention group participated in interventions in the period between T1 and T2, whereas the control group received the same interventions between the T3 and T4 follow‐up measurements. Finally, the number of changes per participant was calculated. Analyses were carried out using R (version 4.5.1; R Core Team 2020).

## 3 Results

The number of participants indicating lifetime prevalence of NSSI at each time point, both out of all those present at each measurement and out of those with five complete measurements, are presented in Table 2. Generally, reported lifetime prevalence decreased over time with 18.90% and 12.60% acknowledging a history of NSSI at T1 and T5, respectively, in the sample with complete measurements at all five time points. Similarly, in participants with incomplete data for one or several of the time points, 20.55% and 14.72% acknowledged lifetime NSSI history at T1 and T5.

**TABLE 2:** Number and percentage of participants acknowledging lifetime history of NSSI at each time point, out of all present participants and out of those with five complete measurements.

	All participants present, per measurement time point			Participants with five complete measurements (*n* = 127)	
	*n* present	*n* “Yes”	%	*n* “Yes”	%
T1	253	52	20.55	24	18.90
T2	201	43	21.39	27	21.26
T3	230	34	14.78	19	14.96
T4	205	30	14.68	20	15.75
T5	197	29	14.72	16	12.60

Abbreviation: NSSI = Nonsuicidal self‐injury.

Out of the 253 participants who answered the single‐item NSSI question at baseline (T1) and at least one follow‐up measurement (T2, T3, T4 or T5), 48 (18.97%) changed their response. Of all participants, 201 (79.45% of the whole sample) answered “No” at baseline. In this subset, 18 (8.96% of the subset) later answered “Yes” at one or more follow‐up measurements, while 183 (91.04%) did not change their answer. In contrast, 52 participants (20.55% of the whole sample) answered “Yes” at baseline, indicating lifetime NSSI history. Of these, 30 (57.69% of the subset) subsequently answered “No”, at a later time point, whereas 22 (42.31%) did not change their response (Table 3). In total, 70 participants responded “Yes” to any of the NSSI measurements. Of these, 39 (55.71%) participants changed their response to “No” at a later time point.

**TABLE 3:** Subgroups of participants' response patterns on lifetime NSSI, based on baseline response.

Group	*n*	% of the full sample (*N* = 253)
Only “No”	183	72.33
Only “Yes”	22	8.70
Baseline “Yes”, then “no” at least once	30	11.86
Baseline “No”, then “yes” at least once	18	7.11

Abbreviation: NSSI = Nonsuicidal self‐injury.

Out of the 52 participants who acknowledged a history of NSSI at baseline (T1), six (11.54%) were male. All six male participants (100%) later changed their response, denying lifetime prevalence of NSSI at a later time point. Among girls initially acknowledging a history of NSSI, 23 out of 40 (57.5%) changed to denying lifetime history of NSSI at a later time point. The same was true for one out of six participants (16.67%) identifying as non‐binary. For gender‐separate reliability statistics, see Tables S1 and S2 in Supporting Information S1.

Calculation of Fleiss' kappa for the 127 participants who had complete data from all five measurements resulted in *κ* = 0.626 (95% CI [0.571, 0.681], *p* < 0.0001), indicating a moderate agreement across time points. After excluding participants who initially responded “No” and later changed to “Yes”, which could be considered “true” and possible changes, 123 complete cases remained, resulting in a slightly improved kappa, *κ* = 0.654 (95% CI [0.598, 0.710], *p* < 0.0001).

Calculation of Cohen's kappa between baseline and 12‐month measurements for the 205 participants who completed both time points resulted in *κ* = 0.588 (95% CI [0.441, 0.735], *p* < 0.0001), indicating moderate agreement. Exclusion of participants who initially responded “No” and changed to “Yes” at 12 months resulted in 198 complete cases remaining, which resulted in a slightly higher kappa of *κ* = 0.683 (95% CI [0.547, 0.820], *p* < 0.0001), indicating substantial agreement.

While the majority of participants present for all five measurements (78.74%) did not change their answer, one in five (21.26%) did. Additionally, some participants (9.45%) changed their response more than once (see Table 4).

**TABLE 4:** Number and percentage of participants per number of changes (count) to responses on the lifetime NSSI question.

Count	Participants with five complete measurements (*n* = 127) *n* (%)
0	100 (78.74)
1	15 (11.81)
2	9 (7.09)
3	3 (2.36)
4	0

As an intervention was delivered, it was necessary to investigate whether the intervention was associated with changes in participants' responses on the single‐item question on NSSI history, as such changes would not be related to reliability. Participants either received the intervention in the period between baseline and three months (T1‐T2 for the intervention group) or between six and 12 months (T3‐T4 for the control group). Chi‐square tests were conducted to compare the number of changes between baseline and three months (T1‐T2), baseline and six months (T1‐T3), and six and 12 months (T3‐T4) across intervention and control groups, in order to investigate whether participants changed their responses to the lifetime NSSI question as an effect of receiving interventions. Results indicated that the number of changes in self‐reported lifetime NSSI history did not differ significantly between groups in the first (*χ* ^2^ = 0.13, *p* = 0.717), second (*χ* ^2^ = 1.07, *p* = 0.302), or third period (*χ* ^2^ = 0.01, *p* = 0.92).

## 4 Discussion

Single‐item measurement of NSSI is commonly used in research to estimate NSSI prevalence. It is also used for screening in clinical settings. Hence, the reliability of single‐item measurement is of high importance. The present study is the first to investigate the performance of single‐item survey measurement of NSSI in adolescents across time. A majority of the participants (91%) who did not acknowledge lifetime history of NSSI did so consistently and did not change their answer at any of the later four time points. However, out of the 52 (21%) participants who acknowledged lifetime history of NSSI (i.e., answering “Yes” to the single‐item question) at T1, a considerable proportion (58%) changed their response from “Yes” to “No” at later time points. Only six male participants initially answered “Yes”, however all (100%) of them eventually changed their answer at a later time point. Reliability calculations indicate that single‐item measurement performs only moderately well over time. Further, the fact that a majority of participants who initially acknowledged NSSI prevalence eventually switched to a negative answer, points to the relatively low stability of “Yes” answers compared to “No” in this community sample of adolescents. This raises the question of how answers regarding lifetime prevalence of NSSI in surveys should be understood and interpreted.

Previous studies have compared the performance of single‐item questions to that of behavioral checklists using cross‐sectional designs have shown that single‐item methodology likely results in false negatives (Robinson and Wilson 2020). Further, findings indicate that single‐item methodology also leads to a biased identification of a group with history of NSSI, as the group acknowledging NSSI differs systematically compared to the group identified using a behavioral checklist, identifying NSSI to a larger degree in girls engaging in common self‐injurious behaviors like cutting or scratching (Aspeqvist et al. 2024).

Several mechanisms have been suggested to be associated with inconsistencies in psychiatric prevalence measures. One such mechanism, reframing, has to do with thinking differently about an experience at a later time, which might lead to a false negative or positive answer at the time of measurement (Streiner et al. 2009). Forgetting is another factor, and Streiner et al. (2009) point to studies indicating that a surprisingly large share of health‐related life events are in fact forgotten after a relatively short time has passed, which is a challenge with retrospective recall. Robinson and Wilson (2020) investigated the hypothesis that behavioral checklists might yield higher prevalence rates as the checklist items, being examples of specific behaviors, might serve as memory cues, and found this not to be the case. Further, it has been suggested that both reframing and forgetting might be factors influencing prevalence rates. Adolescents acknowledging having “hurt themselves on purpose” sometimes include behaviors outside of the scientific definition of NSSI (Ross and Heath 2002) which might be more susceptible to reframing.

It has been suggested that participants might interpret a single‐item question as whether they are more or less similar to individuals engagning in NSSI (Swannell et al. 2014), leading to participants' responses being influenced by their subjective identity. Experiencing identity confusion—a less coherent sense of how one's identity is defined—during adolescence has been linked to increased risk of engaging in NSSI (Bakali Tahiri et al. 2025). This opens to the suggestion that adolescents with a history of NSSI could experience fluctuating identities to a larger degree, including identifying or not as someone engaging in NSSI.

Analyses did not indicate that intervention exposure affected participants' response changes. However, it is worth noting that the overall percentage of participants acknowledging NSSI differed across measurement time points, with the lowest percentage observed at the final measurement. This could hypothetically be a explained by a delayed effect of learning about NSSI, but it could also be related to the above mentioned mechanisms, such as reframing and forgetting.

Taken together, results from this study align with what has been suggested in previous research (Aspeqvist et al. 2024; Robinson and Wilson 2020; Swannell et al. 2014), that is, that subjective identification could play a role in adolescents' responses to questions on lifetime NSSI. Single‐item assessment is most probably processed by the semantic memory system, touching on implicit identification (Nock 2010), such as “I am someone who self‐injurs”. Such identification might be subject to change over time, both as weekly or monthly fluctuation and as changing with development during adolescence, thus explaining the instability in participants' response pattern. This might be one reason why the single‐item questions is acknowledged to a larger degree by girls than boys compared to behavioral checklists (Aspeqvist et al. 2024). It is also possible that other factors, such as the current emotional state experienced during the time of the survey, influences reporting and could lead to skewed results. There is of course also the possibility that some adolescents respond to survey items without putting much thought into the process, which could result in inconsistent responses during different time points.

Findings in the present study indicate that adolescents' self‐reported responses to single‐item survey questions concerning lifetime NSSI history ideally need to be validated against other sources of information, such as checklists and interviews to ensure that adolescents' current interpretations and perspectives of the screening item are understood correctly. This seems to apply especially to “Yes” answers, as results show that slightly more than half of participants acknowledging lifetime prevalence of NSSI with single‐item at some later time point answered “No” to the same question. The findings are relevant beyond survey‐based epidemiology, as structured interviews used in both research and clinical settings may also use respondents' answers to an initial, dichotomous question on lifetime NSSI to determine whether to proceed to further questions on NSSI or skip to the next section.

In sum, several potential mechanisms can lead to less reliable estimates over time of the single‐item screening assessment, implicating that caution must be taken when interpretating cross‐sectional survey results.

### 4.1 Limitations

While the use of a longitudinal dataset to evaluate responses to NSSI lifetime prevalence in a community sample of adolescents is a strength of the present study, it also has some limitations. Selection bias is an obvious challenge to the generalizability of the study results, as only 25.3% of eligible adolescents participated. Further, attrition bias might be influencing the results, as 253 out of 266 (95.11%) participated in data collection after baseline and only 127 (47.74%) had data from all five measurement points. A few participants changed their response from “No” to “Yes” at some point from T1 to T5, which might indicate having engaged in NSSI for the first time. This is reasonable given the increase in NSSI onset over time during ages 14–16 years (Plener et al. 2015). For this reason, separate analyses were carried out excluding this subgroup. One limitation that needs to be taken into account when interpreting the results of the current study, is that an intervention was conducted in between measuring points, which could potentially influence how NSSI was conceptualized and viewed, leading to different responses on the single‐item. Our results did not confirm this assumption, however, as analyses of the effect of the intervention on the single‐item measure showed non‐significant results.

### 4.2 Conclusions

Results from the present study point to the moderate reliability of single‐item NSSI lifetime prevalence survey questions. This seems to be especially true for answers acknowledging NSSI. Researchers and clinicians need to be aware of the limitations of screening with single‐item as a sole method. The complexity of measuring behaviors with self‐report argue for the need to calibrate response patterns with other data sources in order to better understand the actual lifetime prevalence of NSSI in adolescents. Larger longitudinal samples would open up the investigation of other factors such as gender, present emotional state, and self‐injury functions and characteristics which might potentially influence participants' responses.

## Author Contributions

**Erik Aspeqvist:** conceptualization, investigation, data curation, formal analysis, writing – original draft, writing – review and editing. **Hedvig Andersson:** conceptualization, investigation, data curation, writing – review and editing. **Örjan Dahlström:** formal analysis, supervision, writing – review and editing. **Laura Korhonen:** conceptualization, supervision, writing – review and editing. **Maria Zetterqvist:** conceptualization, funding acquisition, supervision, writing – review and editing.

## Funding

This work was supported by the Swedish Research Council (2018–05820).

## Conflicts of Interest

The authors declare no conflicts of interest.

## Supporting information


Supporting Information S1

## Data Availability

The datasets generated and analyzed during the current study are not publicly available due to limitations written into the participants' consent forms. Group level data are available from the corresponding author on reasonable request.

## References

[mpr70115-bib-0001] Aspeqvist, E. , H. Andersson , L. Korhonen , Ö. Dahlström , and M. Zetterqvist . 2024. “Measurement and Stratification of Nonsuicidal Self‐Injury in Adolescents.” BMC Psychiatry 24, no. 1: 107. 10.1186/s12888-024-05535-3. PMC10848387 38326791

[mpr70115-bib-0002] Aspeqvist, E. , L. Korhonen , O. Dahlstrom , et al. 2025. “Effects of a Whole‐School Prevention Program Targeting Mental Health and Nonsuicidal Self‐Injury in Swedish Adolescents: A Cluster‐Randomized Experimental Study With Longitudinal Follow‐Up.” Journal of Youth and Adolescence 55, no. 4: 795–813. 10.1007/s10964-025-02251-3. PMC13076508 40932580

[mpr70115-bib-0003] Bakali Tahiri, M. J. , L. Vankerckhoven , L. Claes , and K. Luyckx . 2025. “Identity, Psychological Distress, and Non‐Suicidal Self‐Injury in Adolescents: Directionality of Effects and Mediational Pathways.” Identity: 1–21. 10.1080/15283488.2025.2604073.

[mpr70115-bib-0004] Borschmann, R. , J. T. Young , P. Moran , et al. 2017. “Accuracy and Predictive Value of Incarcerated Adults' Accounts of Their Self‐Harm Histories: Findings Froman Australian Prospective Data Linkage Study.” CMAJ Open 5, no. 3: E694–E701. 10.9778/cmajo.20170058. PMC5621944 28893844

[mpr70115-bib-0005] Cohen, J. 1960. “A Coefficient of Agreement for Nominal Scales.” Educational and Psychological Measurement 20, no. 1: 37–46. 10.1177/001316446002000104.

[mpr70115-bib-0006] Daukantaite, D. , R. Lantto , S. I. Liljedahl , M. Helleman , and S. Westling . 2020. “One‐Year Consistency in Lifetime Frequency Estimates and Functions of Non‐Suicidal Self‐Injury in a Clinical Sample.” Frontiers in Psychiatry 11: 538. 10.3389/fpsyt.2020.00538. PMC7308529 32612546

[mpr70115-bib-0007] Daukantaite, D. , L. G. Lundh , M. Wangby‐Lundh , et al. 2020. “What Happens to Young Adults who Have Engaged in Self‐Injurious Behavior as Adolescents? A 10‐Year Follow‐Up.” European Child & Adolescent Psychiatry 30, no. 3: 475–492. 10.1007/s00787-020-01533-4. PMC8019412 32318877

[mpr70115-bib-0008] DeVille, D. C. , D. Whalen , F. J. Breslin , et al. 2020. “Prevalence and Family‐Related Factors Associated With Suicidal Ideation, Suicide Attempts, and Self‐Injury in Children Aged 9 to 10 Years.” JAMA Network Open 3, no. 2: e1920956. 10.1001/jamanetworkopen.2019.20956. PMC7261143 32031652

[mpr70115-bib-0009] Fleiss, J. L. 1971. “Measuring Nominal Scale Agreement Among Many Raters.” Psychological Bulletin 76, no. 5: 378–382. 10.1037/h0031619.

[mpr70115-bib-0010] Gillies, D. , M. A. Christou , A. C. Dixon , et al. 2018. “Prevalence and Characteristics of Self‐Harm in Adolescents: Meta‐Analyses of Community‐Based Studies 1990–2015.” Journal of the American Academy of Child & Adolescent Psychiatry 57, no. 10: 733–741. 10.1016/j.jaac.2018.06.018. 30274648

[mpr70115-bib-0011] International Society for the Study of Self‐Injury . 2025. What is Self‐Injury?. https://www.itriples.org/aboutnssi/what‐is‐self‐injury.

[mpr70115-bib-0012] Kessler, R. C. , P. Berglund , O. Demler , R. Jin , K. R. Merikangas , and E. E. Walters . 2005. “Lifetime Prevalence and Age‐of‐Onset Distributions of DSM‐IV Disorders in the National Comorbidity Survey Replication.” Archives of General Psychiatry 62, no. 6: 593–602. 10.1001/archpsyc.62.6.593. 15939837

[mpr70115-bib-0013] Kiekens, G. , P. Hasking , M. Boyes , et al. 2018. “The Associations Between Non‐Suicidal Self‐Injury and First Onset Suicidal Thoughts and Behaviors.” Journal of Affective Disorders 239: 171–179. 10.1016/j.jad.2018.06.033. 30014957

[mpr70115-bib-0014] Klonsky, E. D. , and C. R. Glenn . 2009. “Assessing the Functions of Non‐Suicidal Self‐Injury: Psychometric Properties of the Inventory of Statements About Self‐Injury (ISAS).” Journal of Psychopathology and Behavioral Assessment 31, no. 3: 215–219. 10.1007/s10862-008-9107-z. PMC5736316 29269992

[mpr70115-bib-0015] Liu, R. T. 2023. “The Epidemiology of Non‐Suicidal Self‐Injury: Lifetime Prevalence, Sociodemographic and Clinical Correlates, and Treatment Use in a Nationally Representative Sample of Adults in England.” Psychological Medicine 53, no. 1: 274–282. 10.1017/S003329172100146X. PMC10324294 33960286

[mpr70115-bib-0016] Merikangas, K. R. , J. P. He , M. Burstein , et al. 2010. “Lifetime Prevalence of Mental Disorders in U.S. Adolescents: Results From the National Comorbidity Survey Replication‐Adolescent Supplement (NCS‐A).” Journal of the American Academy of Child & Adolescent Psychiatry 49, no. 10: 980–989. 10.1016/j.jaac.2010.05.017. PMC2946114 20855043

[mpr70115-bib-0017] Muehlenkamp, J. J. , L. Claes , L. Havertape , and P. L. Plener . 2012. “International Prevalence of Adolescent Non‐Suicidal Self‐Injury and Deliberate Self‐Harm.” Child and Adolescent Psychiatry and Mental Health 6, no. 1: 10. 10.1186/1753-2000-6-10. PMC3348041 22462815

[mpr70115-bib-0018] Nock, M. K. 2010. “Self‐Injury.” Annual Review of Clinical Psychology 6, no. 1: 339–363. 10.1146/annurev.clinpsy.121208.131258. 20192787

[mpr70115-bib-0019] Nock, M. K. , E. B. Holmberg , V. I. Photos , and B. D. Michel . 2007. “Self‐Injurious Thoughts and Behaviors Interview: Development, Reliability, and Validity in an Adolescent Sample.” Psychological Assessment 19, no. 3: 309–317. 10.1037/1040-3590.19.3.309. 17845122

[mpr70115-bib-0020] Plener, P. L. , T. S. Schumacher , L. M. Munz , and R. C. Groschwitz . 2015. “The Longitudinal Course of Non‐Suicidal Self‐Injury and Deliberate Self‐Harm: A Systematic Review of the Literature.” Borderline Personal Disord Emot Dysregul 2, no. 1: 2. 10.1186/s40479-014-0024-3. PMC4579518 26401305

[mpr70115-bib-0021] R Core Team . 2020. R: A Language and Environment for Statistical Computing.

[mpr70115-bib-0022] Robinson, K. , and M. S. Wilson . 2020. “Open to Interpretation? Inconsistent Reporting of Lifetime Nonsuicidal Self‐Injury Across Two Common Assessments.” Psychological Assessment 32, no. 8: 726–738. 10.1037/pas0000830. 32352812

[mpr70115-bib-0023] Ross, S. , and N. Heath . 2002. “A Study of the Frequency of Self‐Mutilation in a Community Sample of Adolescents.” Journal of Youth and Adolescence 31, no. 1: 67–77. 10.1023/a:1014089117419.

[mpr70115-bib-0024] Staring, L. , G. Kiekens , and O. J. Kirtley . 2023. “The Epidemiology of Nonsuicidal Self‐Injury and Self‐Harm in Nonclinical Samples Population‐Level Trends.” In The Oxford Handbook of Nonsuicidal Self‐Injury, edited by E. E. Lloyd‐Richardson , I. Baetens , and J. L. Whitlock . 10.1093/oxfordhb/9780197611272.013.7.

[mpr70115-bib-0025] Streiner, D. L. , S. B. Patten , J. C. Anthony , and J. Cairney . 2009. “Has 'Lifetime Prevalence' Reached the End of its Life? An Examination of the Concept.” International Journal of Methods in Psychiatric Research 18, no. 4: 221–228. 10.1002/mpr.296. PMC4906789 20052690

[mpr70115-bib-0026] Swannell, S. V. , G. E. Martin , A. Page , P. Hasking , and N. J. St John . 2014. “Prevalence of Nonsuicidal Self‐Injury in Nonclinical Samples: Systematic Review, Meta‐Analysis and Meta‐Regression.” Suicide and Life‐Threatening Behavior 44, no. 3: 273–303. 10.1111/sltb.12070. 24422986

[mpr70115-bib-0027] Wichstrom, T. , and L. Wichstrom . 2024. “Childhood Predictors of Nonsuicidal Self‐Injury in Adolescence: A Birth Cohort Study.” Journal of the American Academy of Child & Adolescent Psychiatry 63, no. 11: 1114–1122. 10.1016/j.jaac.2023.12.013. 38423281

[mpr70115-bib-0028] Xiao, Q. , X. Song , L. Huang , D. Hou , and X. Huang . 2022. “Global Prevalence and Characteristics of Non‐Suicidal Self‐Injury Between 2010 and 2021 Among a Non‐Clinical Sample of Adolescents: A Meta‐Analysis.” Frontiers in Psychiatry 13: 912441. 10.3389/fpsyt.2022.912441. PMC9399519 36032224

[mpr70115-bib-0029] Zetterqvist, M. , L. S. Jonsson , A. Landberg , and C. G. Svedin . 2021. “A Potential Increase in Adolescent Nonsuicidal Self‐Injury During covid‐19: A Comparison of Data From Three Different Time Points During 2011–2021.” Psychiatry Research 305: 114208. 10.1016/j.psychres.2021.114208. PMC8448460 34543849

[mpr70115-bib-0030] Zetterqvist, M. , L. G. Lundh , O. Dahlstrom , and C. G. Svedin . 2013. “Prevalence and Function of Non‐Suicidal Self‐Injury (NSSI) in a Community Sample of Adolescents, Using Suggested DSM‐5 Criteria for a Potential NSSI Disorder.” Journal of Abnormal Child Psychology 41, no. 5: 759–773. 10.1007/s10802-013-9712-5. 23344701

