# Supplementary material for: Lifetime Prevalence of Nonsuicidal Self‐Injury in Adolescents: Reliability of a Dichotomous Survey Item
Source: Int J Methods Psychiatr Res. 2026 Oct 21;35(4):e70115. doi: 10.1002/mpr.70115 (PMC13685060; doi:10.1002/mpr.70115)
Supplement: Supplementary file 1 [file MPR-35-e70115-s001.docx]

# Supplementary Materials

## Gender-specific reliability analyses

| **Table S1**  Fleiss’ kappa for participants with five complete measurements | | | | |
| --- | --- | --- | --- | --- |
| Group | *n* | Kappa | 95% CI | *p* |
| All participants | | | | |
| Female | 74 | 0.573 | 0.501; 0.645 | < 0.0001 |
| Male | 51 | 0.575 | 0.488; 0.662 | < 0.0001 |
| Excluding changes from “no” to “yes” | | | | |
| Female | 71 | 0.611 | 0.538; 0.685 | < 0.0001 |
| Male | 50 | 0.525 | 0.437; 0.612 | < 0.0001 |

*Note*: The number of participants indicating non-binary gender (six) was too low for meaningful reliability analysis.

| **Table S2**  Cohen’s kappa between baseline and twelve months | | | | |
| --- | --- | --- | --- | --- |
| Group | *n* | Kappa | 95% CI | *p* |
| All participants | | | | |
| Female | 124 | 0.546 | 0.368; 0.725 | < 0.0001 |
| Male | 76 | 0.474 | 0.038; 0.910 | < 0.0001 |
| Excluding changes from “no” to “yes” | | | | |
| Female | 118 | 0.661 | 0.498; 0.825 | < 0.0001 |
| Male | 75 | 0.554 | 0.112; 0.997 | < 0.0001 |

*Note*: The number of participants indicating non-binary gender (six) was too low for meaningful reliability analysis.
